# Supplementary material for: Quality Evaluation of Dietary Supplements for Weight Loss Based on Garcinia cambogia
Source: Nutrients. 2022 Jul 27;14(15):3077. doi: 10.3390/nu14153077 (PMC9332841; doi:10.3390/nu14153077)
Supplement: Supplementary file 1 [file nutrients-14-03077-s001.zip › nutrients-1807929-supplementary.pdf]

Table S1. Characteristics and composition (data per formulation unit) declared on labels of *G. cambogia* food supplements under study.

Code	Type	*G. cambogia* amount ((-)-HCA content)	Other declared constituents	Acquisition
GCFS1A*	Tablets	416 mg *G. cambogia* (≥ 60% (-)-HCA)	20.625 mg choline, 0.35 mg vitamin B6, 20 mg vitamin C, 10 µg chrome	Herbal shop
GCFS1B*	Tablets	416 mg *G. cambogia* (≥ 60% (-)-HCA)	20.625 mg choline, 0.35 mg vitamin B_6, 0.20 mg vitamin C, 10 µg chrome	Herbal shop
GCFS2	Capsules	250 mg *G. cambogia* extract (60% (-)-HCA)	-	Herbal shop
GCFS3	Capsules	401 mg *G. cambogia* extract (50% (-)-HCA)	-	Pharmacy
GCFS4	Capsules	350 mg *G. cambogia* dry extract (50% (-)-HCA)	60 mg vitamin C, 40 µg chrome	Supermarket
GCFS5	Capsules	350 mg *G. cambogia* fruit dry extract (175 mg (-)-HCA)	Maltodextrin, magnesium stearate	Supermarket
GCFS6	Capsules	300 mg *G. cambogia* dry extract (60 % (-)-HCA, 180 mg)	Cellulose	Online
GCFS7	Capsules	300 mg Garcinia dry extract (60 % (-)-HCA)	Mannitol, cellulose, magnesium stearate and silicon dioxide	Online
GCFS8	Capsules	500 mg *G. cambogia* fruit dry extract (60% (-)-HCA)	Magnesium stearate and silicon dioxide	Supermarket
GCFS9	Tablets	1200 mg *G. cambogia* fruit dry extract (60% (-)-HCA, 720 mg)	Magnesium stearate and cellulose, cornstarch	Herbal shop
GCFS10	Capsules	1500 mg *G. cambogia* fruit extract (60% (-)-HCA)	Cellulose, magnesium stearate, silicon dioxide	Online
GCFS11	Tablets	Garcinia dry extract (fruit) (60% (-)-HCA, 360 mg)	Cellulose, maltodextrins, calcium phosphate, magnesium stearate	Online
GCFS12	Capsules	500 mg *G. cambogia* fruit rind extract (60% (-)-HCA)	Magnesium stearate, silicon dioxide	Online
GCFS13	Capsules	1000 mg *G. cambogia* extract	Hydroxypropylmethyl cellulose, Magnesium stearate	Online
GCFS14	Capsules	500 mg *G. cambogia* pure extract	Cellulose, magnesium stereate, silicon dioxide	Online
GCFS15	Tablets	2000 mg per 2 caplets, *G. cambogia* fruit dry extract. (60% (-)-HCA, 1200 mg per 2 caplets)	Sodium carboxymethyl cellulose, cellulose, dicalcium phosphate, magnesium stereate, silicon dioxide	Online
GCFS16	Capsules	*G. cambogia* 4:1, pure natural source (60% (-)-HCA)	Calcium carbonate, hydroxyprophylmethyl cellulose	Online
GCFS17	Powder	*G. cambogia* dry extract 61.60% (-)-HCA	-	Biotechnological company

*Supplements from the same brand but of different production batches

Table S2. Analytical parameters for the validation of the LC-UV method for (-)-HCA determination (*n* = 5).

Calibration curve	y = 146147377x – 1234424 (R^2 = 0.9991)
Linear range (mg mL^{-1})	0.05–1
Repeatability (RSD %)	1.45
Intermediate precision (RSD %)	1.17
LOD (mg mL^{-1})<br>*LOQ* (mg mL^{-1})	0.140 (SD 0.005)<br>0.45 (SD 0.015)
Recovery (%)<br>GFS12 + 0.05 mg<br>GFS12 + 0.15 mg<br>GFS12 + 0.25 mg	 100.9 (SD 1.3)<br>101.2 (SD 2.4)<br>101.3 (SD 1.7)

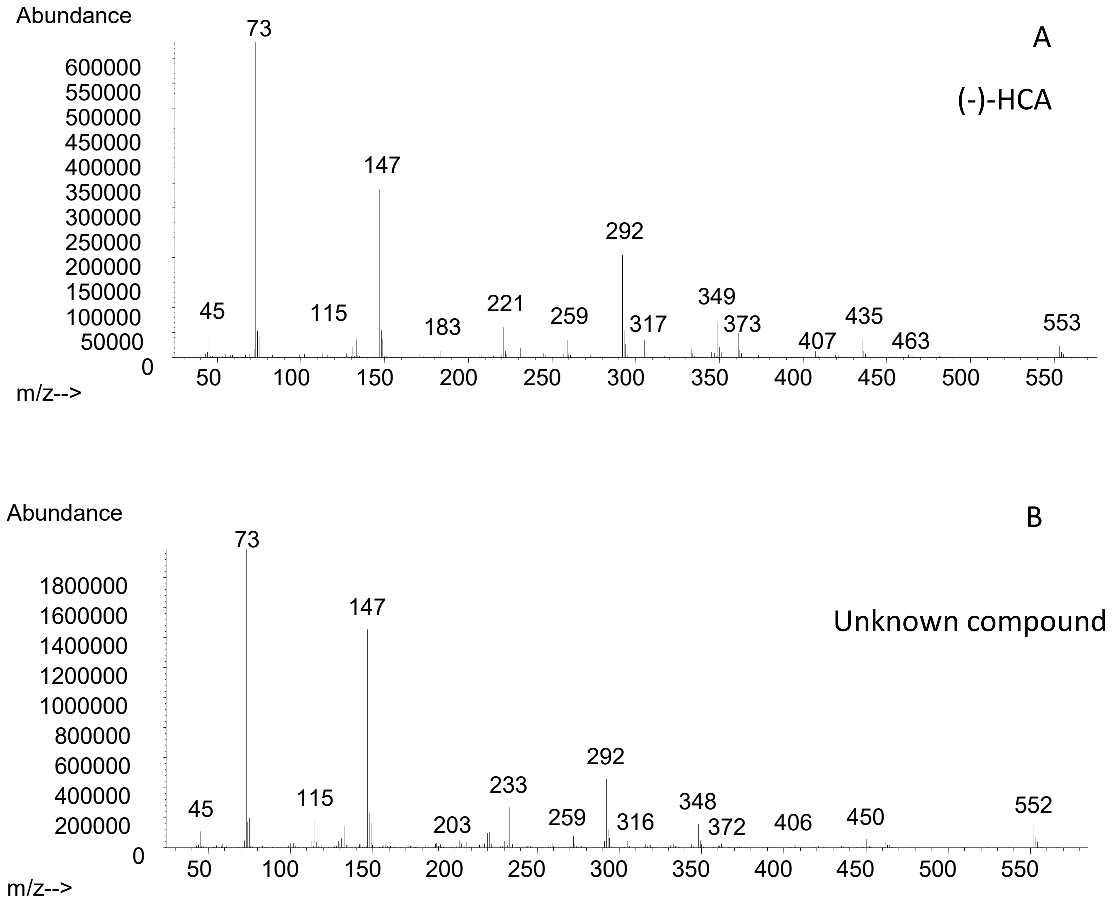

Figure S1. Mass spectra of derivatized (-)-HCA (**A**) and an unknown compound (**B**) eluting by gas chromatography at t_R 9.63 min found in kudam puli extracts.
